# Plasma Glutamine Concentrations in Liver Failure

**DOI:** 10.1371/journal.pone.0150440

**Published:** 2016-03-03

**Authors:** Gunnel Helling, Staffan Wahlin, Marie Smedberg, Linn Pettersson, Inga Tjäder, Åke Norberg, Olav Rooyackers, Jan Wernerman

**Affiliations:** 1 Department of Anesthesia & Intensive Care, Karolinska University Hospital Huddinge and Karolinska Institutet, Stockholm, Sweden; 2 Department of gastroenterology and hepatology, Karolinska University Hospital and Karolinska Institutet, Stockholm, Sweden; Kaohsiung Chang Gung Memorial Hospital, TAIWAN

## Abstract

**Background:**

Higher than normal plasma glutamine concentration at admission to an intensive care unit is associated with an unfavorable outcome. Very high plasma glutamine levels are sometimes seen in both acute and chronic liver failure. We aimed to systematically explore the relation between different types of liver failure and plasma glutamine concentrations.

**Methods:**

Four different groups of patients were studies; chronic liver failure (n = 40), acute on chronic liver failure (n = 20), acute fulminant liver failure (n = 20), and post-hepatectomy liver failure (n = 20). Child-Pugh and Model for End-stage Liver Disease (MELD) scores were assessed as indices of liver function. All groups except the chronic liver failure group were followed longitudinally during hospitalisation. Outcomes were recorded up to 48 months after study inclusion.

**Results:**

All groups had individuals with very high plasma glutamine concentrations. In the total group of patients (n = 100), severity of liver failure correlated significantly with plasma glutamine concentration, but the correlation was not strong.

**Conclusion:**

Liver failure, regardless of severity and course of illness, may be associated with a high plasma glutamine concentration. Further studies are needed to understand whether high glutamine levels should be regarded as a biomarker or as a contributor to symptomatology in liver failure.

## Introduction

A low plasma concentration of glutamine is seen in around 30% of ICU admission, and is an independent predictor for mortality [1,2]. Also above-normal-range plasma glutamine concentrations are associated with an unfavorable outcome [2]. However, in a mixed population of ICU admissions, patients with high glutamine levels are much fewer than those with low values [3,4]. Patients with liver failure seem to be overrepresented among these 5–10% with high levels [2].

Patients with acute fulminant liver failure are known to have high or even very high plasma glutamine levels [5]. High glutamine levels have been suggested to be related to high ammonia levels and to the development of hepatic encephalopathy [6,7]. Most reports have associated glutamine with encephalopathy in acute fulminant liver failure, while the relation between high glutamine levels and encephalopathy in acute-on chronic liver failure is less well described [6,8,9]. Oral glutamine boluses to assess the susceptibility for encephalopathy has been suggested [10]. In a comparative study, acute fulminant liver failure was associated with high plasma glutamine levels, but chronic or acute-on-chronic liver failure were not [5].

Acute fulminant liver failure is therefore regarded a contraindication for glutamine substitution during critical illness [11,12]. It is less clear if patients with chronic and acute-on-chronic liver failure should also be exempted from glutamine supplementation. These patients are often malnourished and therefore at particular risk for nutrition-related complications.

In order to clarify glutamine status in patients with liver failure, four different groups of patients were studied descriptively. Patients with (A) chronic liver failure, (B) acute-on-chronic liver failure, (C) acute fulminant liver failure, and (D) post-hepatectomy liver failure. The latter three groups were also studied longitudinally during hospital stay.

## Materials and Methods

### Patients

Four different groups of adult (18 years or older) patients with liver failure were prospectively included. Absence of informed consent or presence of a do-not-resuscitate order were the only exclusion criteria. A. Patients with chronic liver failure (n = 40) during a planned appointment to the hepatology out-patient clinic. B. Patients with acute-on-chronic liver failure (n = 20) admitted to the ICU. C. Patients with acute fulminant liver failure (n = 20) admitted to the ICU. D. Patients undergoing elective major liver resections (n = 20). The latter patients were included when postoperatively transferred to the high dependency ward. Before obtaining patients’ written informed consent, patients (or next of kin) were informed verbally and in writing about the study protocol and possible risks involved. The study was carried out in accordance with the guidelines of the Helsinki declaration. The Regional Ethical Review Board in Stockholm (http://www.epn.se/en) approved the study, Dnr 2009–1303.

### Protocol

At the time of blood sampling, liver function was evaluated by Child-Pugh scoring and MELD (Model for End-stage Liver Disease) scoring [13,14]. Group A, chronic liver failure, was only sampled and evaluated once in the out-patient clinic. The 3 other groups were sampled and evaluated first on the day of ICU admission (groups B and C) or at the recovery room (group D), and thereafter every 3^rd^ day (±1 day) during their hospital stay. Number of patients in the 4 groups was a pragmatic decision related to low incidence of in particular patients with acute fulminant liver failure, where less than 20 subjects was considered insufficient. Any intravenous glutamine supplementation during ICU admission was recorded. Liver transplantations were recorded and mortality data were acquired through the Swedish national population registry. Patient management and treatment followed standard hospital protocols.

### Feeding

Patients in groups B and D admitted to the surgical high dependency unit or the ICU were fed according to standard hospital guidelines. Enteral nutrition was the primary choice. If measured by indirect calorimetry, nutrition was dosed in accord with measured energy expenditure; otherwise caloric target was 20 kcal/kg/day. Standard nutrition products were used. Complementary parenteral nutrition was sometimes used. The CRF only collected information about whether or not IV glutamine supplementation was given or not during ICU stay.

### Analysis

After blood sampling in EDTA vials, kept on ice less than 30 minutes, centrifugation at +4°C to obtain plasma and thereafter stored at -80°C pending analysis. Plasma glutamine concentration was analyzed by high pressure liquid chromatography (HPLC) using an on-column derivatization with ortho-phtaldialdehyde/3-mercaptopropionic acid (OPA/3-MPA) as described earlier [15]. The reference interval, 400–930 μmol/L, was defined as the concentration interval at ICU admissions not associated with unfavorable outcomes in consecutive patients [2]. It is almost identical to the concentrations interval seen in healthy individuals [16].

### Statistics

Data are presented as median (range). Spearman’s rank correlation (*r*_*s*_) was used to compare plasma glutamine concentrations with indices of liver function, and receiver operating characteristic curves (ROC curves) were generated by logistic regression using the software STATISTICA 10, (StatSoft, Inc. Tulsa, OK).

## Results

**Fig 1:**
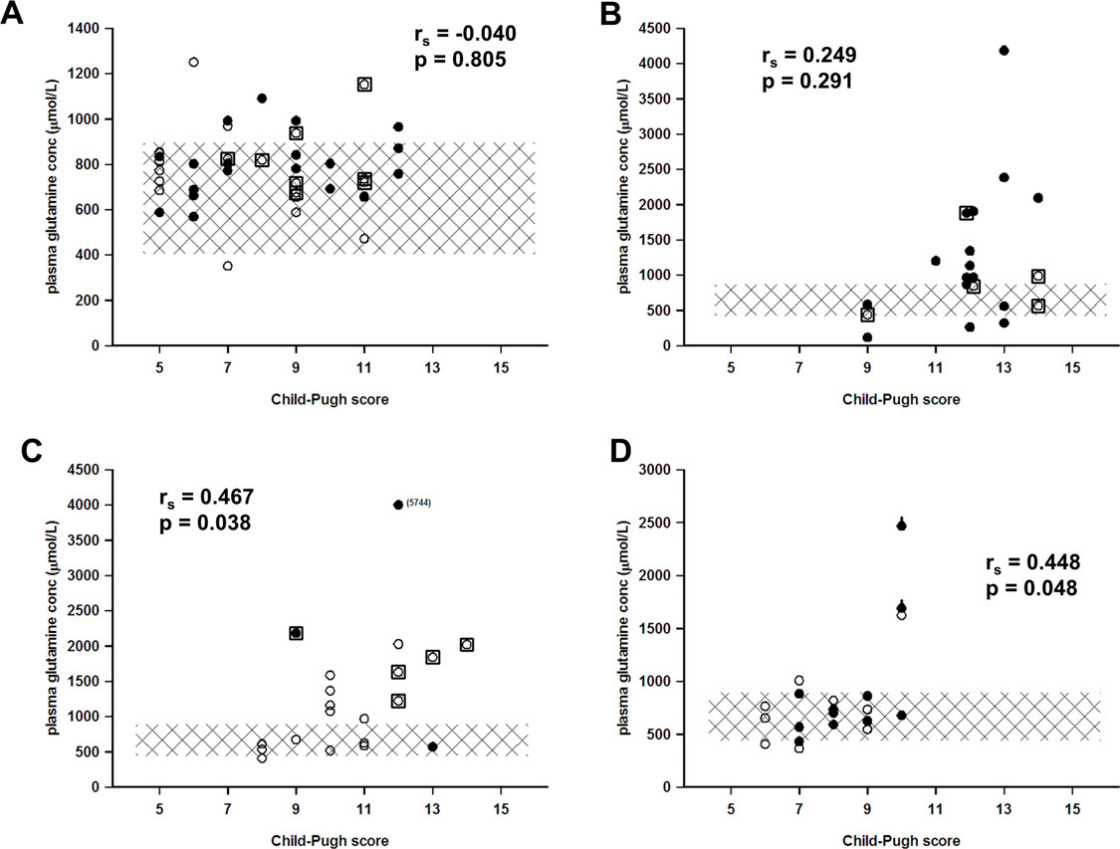
Plasma glutamine concentration in relation to the Child-Pugh score as a reflection of the degree of liver failure. In panel A chronic liver failure (n = 40), in panel B acute-on-chronic liver insufficiency (n = 20), in panel C acute fulminant liver failure (n = 20), and in panel D on the second postoperative day following major liver resection (n = 20). Open round symbols denote alive and filled round symbols denote dead 48 months after sampling. A thick sign on symbols in panel D denotes mortality in the acute phase. Squared inclusion denotes liver transplanted. The hatched area corresponds to the reference interval for plasma glutamine concentration. The r_s_-values for Spearman’s rank correlations together with the P-values for statistical significance are included in all four panels. Observe the different scales of the y-axes.

**Fig 2:**
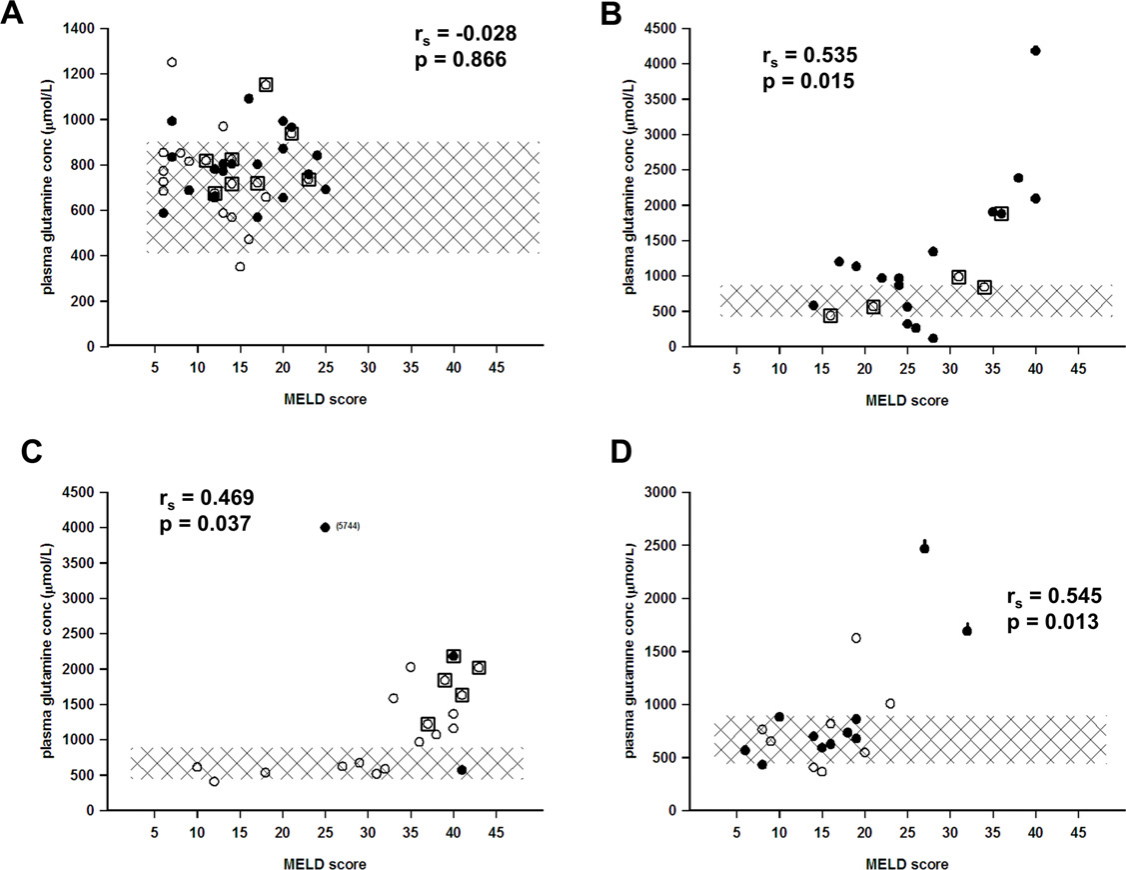
Plasma glutamine concentration in relation to the MELD score as a reflection of the degree of liver failure. In panel A chronic liver failure (n = 40), in panel B acute-on-chronic liver failure (n = 20), in panel C acute fulminant liver failure (n = 20), and in panel D on the second postoperative day following major liver resection (n = 20). Open round symbols denote alive and filled round symbols denote dead 48 months after sampling. Squared inclusion denotes liver transplanted. A thick sign on symbols in panel D denotes mortality in the acute phase. The hatched area corresponds to the reference interval for plasma glutamine concentration. The r_s_-values for Spearman’s rank correlations together with the P-values for statistical significance are included in all four panels. Observe the different scales of the y-axes.

**Fig 3:**
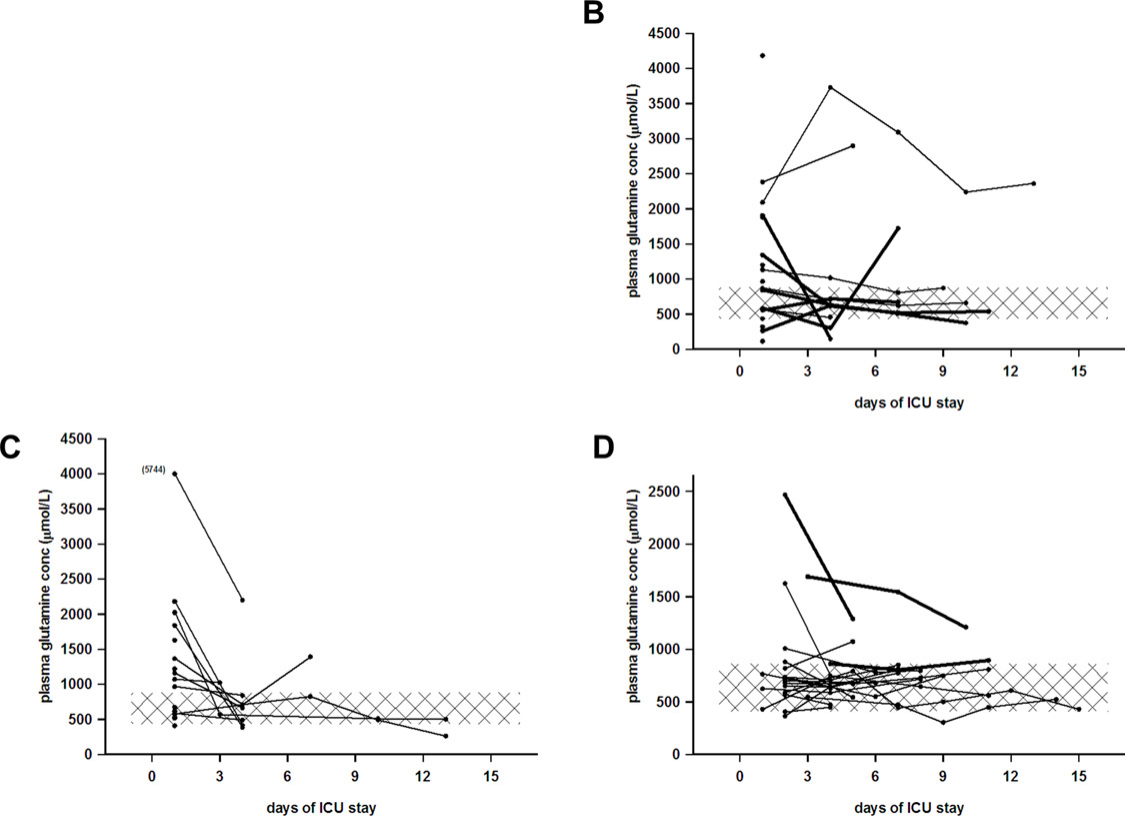
**Longitudinal plasma glutamine concentration during hospital stay** (or until acute liver transplantation in panel C) in panel B acute-on-chronic liver failure (n = 20), in panel C acute fulminant liver failure (n = 20), and in panel D following major liver resection (n = 20). Bold lines denote ongoing IV glutamine supplementation whilst staying in the ICU. Group A, chronic liver failure, was not followed longitudinally. Observe the different scales of the y-axes. In Group B, acute on chronic liver failure (n = 20), the decompensation etiologies or reasons for ICU admission were: gastrointestinal bleeding (n = 8), severe sepsis/septic shock (n = 5), spontaneous bacterial peritonitis (n = 3), hepatorenal syndrome (n = 2), and encephalopathy West-Haven grade 4 (n = 2). Plasma glutamine concentration at ICU admission was 968 (115–4,184) μmol/L. Three patients were below and 11 patients were above the reference interval of 400–930 μmol/L. In Group C, acute fulminant liver failure (n = 20), the etiologies of liver failure were paracetamol intoxication (n = 9), ischemia (n = 4), acute viral hepatitis (n = 2), mushroom poisoning (n = 1), Wilson’s disease (n = 1), and unknown (n = 3). Plasma glutamine concentration at hospital admission was 1,116 (411–5,744) μmol/L. No patient was below and 11 patients were above the reference interval of 400–930 μmol/L. In Group D, patients undergoing liver resection (n = 20), the indications for liver resection were: metastatic colorectal cancer (n = 14), cholangiocarcinoma (n = 5) and hepatocellular cancer (n = 1). Liver resection was 50–80%, and median peroperative bleeding was 1,800 (400–20,500) mL. Four patients were admitted to the ICU during the postoperative course. Plasma glutamine concentration at transfer from recovery room to high dependency unit on day 1–2 after surgery was 717 (365–2,468) μmol/L. One patient was below and four patients were above the reference interval of 400–930 μmol/L.

The basic characteristics of the patients are presented in Table 1, and the etiologies behind chronic liver failure in Table 2. The Child-Pugh and MELD scores along with outcomes are presented in Figs 1 and 2, and plasma glutamine concentrations over time during hospital stay are presented in Fig 3.

**Table 1:** Characteristics of four groups of patients with liver failure studied for plasma glutamine concentration.

	chronic	acute-on-chronic	acute fulminant	post-hepatectomy
	(n = 40)	(n = 20)	(n = 20)	(n = 20)
gender (male/female)	30/10	11/9	8/12	10/10
age (years)	57.5 (42–75)	60 (25–75)	48 (18–77)	67.5 (51–80)
BMI (kg/m^2^)	26.5 (20–51)	26 (22–40)	24 (20–38)	27 (23–40)
Child-Pugh score	8 (5–12)	12 (9–14)	10.5 (8–13)	8 (6–10)
MELD score	14(6–25)	26 (14–40)	35.5 (10–43)	16 (6–32)
SOFA score	NA	14 (6–19)	7.5 (2–21)	4.5 (1–12)
12-month mortality (%)	47.5	85	15	55
12-month LTx (%)	20	25	25	NA
12-month dead and/or LTx (%)	67.5	100	35	55

Values are given as medians and ranges when applicable. BMI–body mass index, MELD–model for end-stage liver disease, SOFA–sequential organ failure assessment, LTx–liver transplantation, NA–not applicable

In Group A, chronic liver failure (n = 40), plasma glutamine concentration was 777 (351–1,251) μmol/L, one patient was below and 8 patients were above the reference interval of 400–930 μmol/L.

**Table 2:** Etiologies behind chronic liver failure in group A.

alcohol	10
alcohol + HCV	6
alcohol + HCC	1
alcohol + HCV + HCC	1
alcohol + AIH	1
HCV	6
HCV + HCC	2
HCV + HIV	1
HCV + HBV	1
NASH	3
AIH	3
AIH + HCC	1
AIH + PBC	1
cryptogen	2
vascular malformation	1

AIH–Autoimmune hepatitis, HBV–Hepatitis B, HCC–Hepatocellular cancer, HCV–Hepatitis C, HIV–Human immunodeficiency virus disease, NASH–Non-alcohol steato-hepatitis, PBC–Primary biliary cirrhosis.

**Fig 4:**
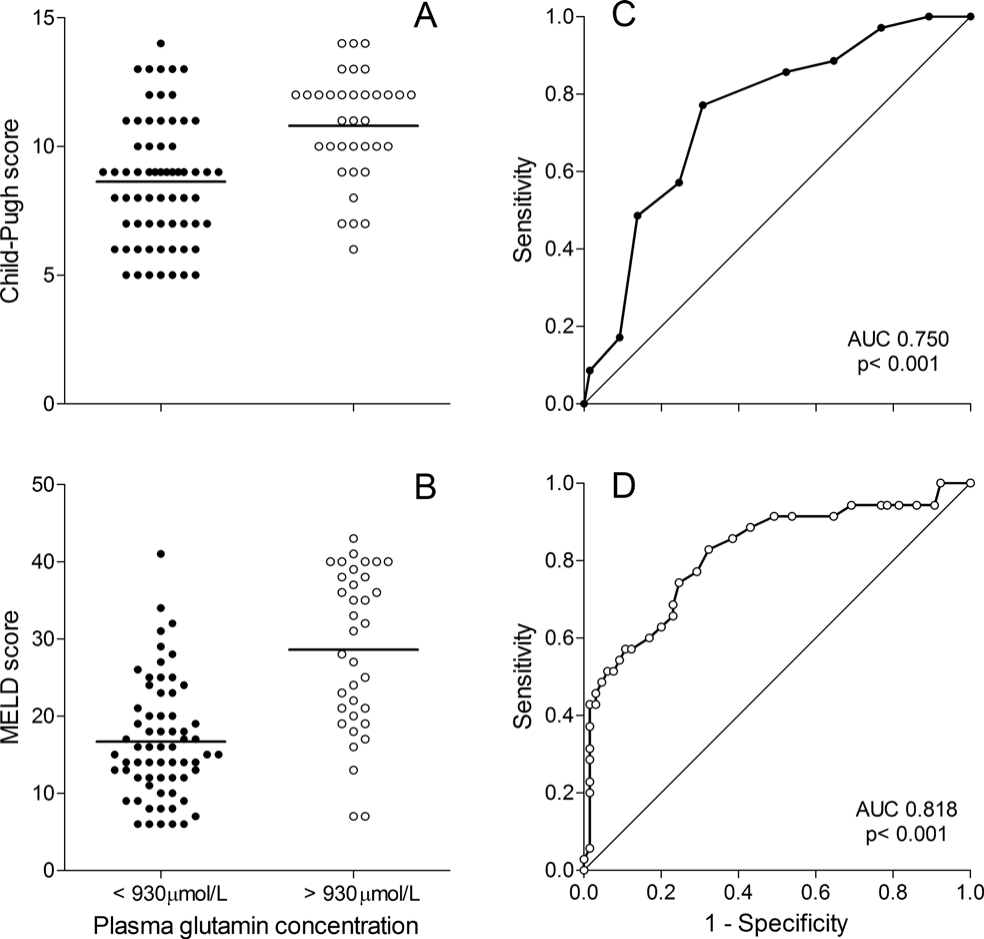
Plasma glutamine concentration dichotomized above or below 930 μmol/L, the upper limit of the reference range, for the total group of patients with liver failure (n = 100). The high out-of-normal–range values were then related to symptoms of liver failure as reflected by the Child-Pugh and MELD scores by receiver operator characteristics curves (ROC curves).

When all four groups of patients (n = 100) were combined, there was a positive statistical rank correlation between the degree of liver failure and plasma glutamine concentration; *r*_*s*_ = 0.32 (p = 0.0014) for Child-Pugh score, and *r*_*s*_ = 0.41 (p = 0.00003) for MELD score. Using a cut-off value of 930 μmol/L for plasma glutamine concentration, receiver operating characteristic curves (ROC curves) were plotted demonstrating the connection between a high out-of-normal range glutamine level and symptoms of liver insufficiency as reflected by Child-Pugh and MELD scores (Fig 4).

## Discussion

There was a positive statistical correlation between degree of liver failure and plasma concentration of glutamine (Fig 4). Although this correlation was highly statistical significant, the correlation was not very strong. Many individual patients deviated from the general trend (Figs 1 and 2), and the four groups of patients exhibited different patterns. All groups contained patients with high or very high plasma glutamine levels, which were associated with a higher degree of liver failure. This study was not designed to elucidate whether higher glutamine concentrations were associated with an increased mortality risks.

The longitudinal follow-up during hospital stay in the three hospitalized groups showed in general terms that patients with high glutamine levels stayed high, and that patients with normal or low levels also stayed in that interval. Even when patients with acute-on-chronic liver failure and patients post liver resection were given IV glutamine supplementation, they remained within respective interval (Fig 3).

The patients with chronic liver failure investigated in the outpatient clinic were mostly in the normal range. Still 20% showed high values that in previous studies have been associated with a higher mortality [2]. For the patients with acute-on-chronic liver failure investigated at ICU admission, 70% showed values previously associated with a higher mortality. In acute-on-chronic liver failure, the majority (55%) had high glutamine concentration and 15% low. In summary patients with chronic liver failure, whether acutely decompensated or not, were normoglutaminemic as a group when there was an indication for ICU admission. Furthermore the clinical relevance of abnormal (high or low) glutamine levels in the acute-on-chronic group of patients needs to be investigated in terms of glutamine production rates, in particular in response to feeding.

The majority (55%) of patients with acute fulminant liver failure showed high initial glutamine concentrations. This group of patients is usually not nutritionally compromised and has little co-morbidity. In relation to symptoms of liver failure, they have a more favorable survival prognosis, although sometimes this involves liver transplantation. This group of patients should not be considered for glutamine supplementation.

In the liver resection group, patients with no history of liver failure were on average older and had much more co-morbidities compared to patients with acute fulminant liver failure. In addition, all patients had an underlying malignancy. Here 25% of patients had high values in conjunction with the acute loss of liver function. Some of these had a complicated course with large per-operative bleedings and postoperative need of ICU admittance. Just one patient showed a low value. As a group, the resected patients were normoglutaminemic and they should therefore also not be considered for glutamine supplementation.

The strength of this study is that the plasma glutamine concentrations presented are combined with classification of patients and characterization of symptoms of liver failure. The main limitation of the study is the non-systematic recruitment of patients, which makes both the connection between glutamine levels and the degree of symptoms of liver failure as well as outcome measures difficult to generalize. Another limitation is the use of Child-Pugh and MELD scoring as measures of the severity of symptoms of liver failure. Although widely used, these scoring systems are only validated for patients with liver cirrhosis.

Among all categories of patients with liver failure included in this study there were individuals with high plasma glutamine concentrations indicative of a high mortality risk in case of ICU admission. Patients with liver failure should not be subjected to exogenous glutamine supplementation unless there is a proven deficiency. More studies are needed to understand the clinical relevance of the out-of-normal-range plasma glutamine concentrations frequently seen in this patient population. In particular, the relation between plasma concentration and the production of glutamine in conjunction with feeding needs to be delineated.

## Conclusion

- High plasma levels of glutamine, common in liver failure, are found to be predictive for an unfavorable outcome in critically ill subjects.
- Here groups of patients with liver failure; chronic, acute-on-chronic, acute fulminant, after extended liver resection, were all found to include subjects with hyperglutaminemia, without relation to severity of illness
- Hyperglutaminemia may just be a biomarker or may have a pathogenetic role related to symptoms associated with liver failure. Further studies are needed to clarify this.
